# Testing for effects of growth rate on isotope trophic discrimination factors and evaluating the performance of Bayesian stable isotope mixing models experimentally: A moment of truth?

**DOI:** 10.1371/journal.pone.0304495

**Published:** 2024-06-14

**Authors:** Kirsty E. B. Gurney, Henry L. Classen, Robert G. Clark

**Affiliations:** 1 Science and Technology Branch, Environment and Climate Change Canada, Saskatoon, Saskatchewan, Canada; 2 College of Agriculture and Bioresources, University of Saskatchewan, Saskatoon, Saskatchewan, Canada; University of Maryland Center for Environmental Science, UNITED STATES

## Abstract

Discerning assimilated diets of wild animals using stable isotopes is well established where potential dietary items in food webs are isotopically distinct. With the advent of mixing models, and Bayesian extensions of such models (Bayesian Stable Isotope Mixing Models, BSIMMs), statistical techniques available for these efforts have been rapidly increasing. The accuracy with which BSIMMs quantify diet, however, depends on several factors including uncertainty in tissue discrimination factors (TDFs; *Δ*) and identification of appropriate error structures. Whereas performance of BSIMMs has mostly been evaluated with simulations, here we test the efficacy of BSIMMs by raising domestic broiler chicks (*Gallus gallus domesticus*) on four isotopically distinct diets under controlled environmental conditions, ideal for evaluating factors that affect TDFs and testing how BSIMMs allocate individual birds to diets that vary in isotopic similarity. For both liver and feather tissues, *δ*^13^C and *δ* ^15^N values differed among dietary groups. *Δ*^13^C of liver, but not feather, was negatively related to the rate at which individuals gained body mass. For *Δ*^15^N, we identified effects of dietary group, sex, and tissue type, as well as an interaction between sex and tissue type, with females having higher liver *Δ*^15^N relative to males. For both tissues, BSIMMs allocated most chicks to correct dietary groups, especially for models using combined TDFs rather than diet-specific TDFs, and those applying a multiplicative error structure. These findings provide new information on how biological processes affect TDFs and confirm that adequately accounting for variability in consumer isotopes is necessary to optimize performance of BSIMMs. Moreover, results demonstrate experimentally that these models reliably characterize consumed diets when appropriately parameterized.

## Introduction

In applications related to trophic ecology, stable isotopes (i.e., the naturally occurring ratio of heavy to light isotopes, primarily for carbon, *δ*^13^C, and nitrogen, *δ*^15^N) are common chemical tracers [1, 2]. Stable isotope analyses are particularly well established for diet reconstruction and expand the scope of inference that can be obtained from other dietary methods, particularly in systems where direct observations are not possible and potential food items are isotopically distinct [3–5]. When metabolically inert tissues such as feathers, hair, or nails are sampled, such methods offer the additional advantage of being non-lethal and less invasive than sampling of other tissues, like blood or muscle [6, 7]. To estimate the proportional contributions of different food resources to consumer tissues, stable isotope analyses primarily use Bayesian stable isotope mixing models (BSIMMs) [8, 9].

Broadly, by using a variety of biological and chemical tracers, statistical models of mixtures (i.e., mixing models) quantify the relative contributions of different source materials to composite, end-member samples [10–12]. Given mathematical limitations of simple linear mixing models, however, BSIMMs, which are flexible and based on a likelihood structure, are most commonly used across the natural sciences to identify proportional inputs of sources to a mixture [13, 14]. Such models incorporate variability into model inputs, allow for inputs from multiple possible sources, and generate potential end-mixture solutions as true probability distributions [15]. Importantly, the Bayesian approach improves upon frequentist methods because processes known to affect the variability in the mixture data are accounted for in model error structures, so that probability distributions reflect associated uncertainties and allow inference about which solutions are more likely than others [16, 17].

The accuracy with which BSIMMs identify source contributions to consumer tissues is strongly influenced by model specifications, especially error assumptions and variation in consumer and resource isotope values related to a suite of factors that can often not be controlled or accounted for [16, 18]. In addition, stable isotope ratios of food items change when incorporated into the tissues of consumers–these changes are described by a trophic discrimination factor (TDF), a key input to BSIMMs that further affects model outputs [19, 20]. Most commonly derived from feeding trials of captive animals, TDFs are highly variable and generally depend on species, diet, and tissue type, although our understanding of how different factors affect TDFs, particularly for carbon, remains limited [21, 22].

To date, most evaluations of how accurately BSIMMs predict diet have been conducted through simulations [16], or controlled studies of captive [19, 23] or wild [5, 24] animals, and reach different conclusions about the relative importance of TDFs on bias, precision, and accuracy of posterior estimates, as well as the factors that affect TDFs. For example, some studies suggest that species-specific TDFs are of secondary importance to the use of informative priors (i.e., existing knowledge about dietary composition) [25], whereas others find that use of an appropriate TDF has a stronger effect on performance of BSIMMs [19, 23, 24]. Further, some data indicate that TDFs vary not only based on nutrient composition of consumed foods and consumer type (i.e., omnivorous, carnivorous), but also on interactions of these factors–and that the factors affecting TDFs vary for different isotopes [22, 26]. An experimental study with tilapia (*Oreochromis mossambicus*) showed the importance of diet type and tissue type in determining TDFs, with simulated data providing additional evidence that the use of experimentally-derived TDFs, compared to those from the literature, improved accuracy of dietary estimates [19]. This is similar to Stephens et al.’s (2022) findings that using existing data and phylogenetic relatedness (i.e., Stable Isotope Discrimination Estimation in R; SIDER) [27] to derive TDFs can lead to misleading conclusions about diet, but is in contrast to other studies that suggest such methods improve BSIMMs performance, or that TDFs affect model results less than other factors [28, 29]. Reliable information about factors affecting TDFs is needed to resolve these apparently contrasting results and to more clearly understand the true, best-case accuracy of BSIMMs outputs in trophic ecology, as well as in other applications [30, 31].

To identify the most critical factors to consider in applying BSIMMs and thus improve inference related estimates of animal diet, we aim to test for factors that affect TDFs and to evaluate how different sources of variation in isotope values drive uncertainty in BSIMM outputs. We test the performance of BSIMMs experimentally, by feeding domestic broiler chicks (*Gallus gallus domesticus*) isotopically distinct diets under controlled environmental conditions, asking three key research questions: (i) how does variation among individual growth rates affect TDFs for carbon (*Δ*^13^C) and nitrogen (*Δ*^15^N) in an omnivorous bird; (ii) how do variable estimates of TDFs and different estimates of uncertainty in BSIMMs influence model performance; and (iii) what is the best-case accuracy of BSIMM outputs when environmental and resource-related properties are primarily controlled for? Although broiler chickens have undergone extensive artificial selection for growth in captivity, variation in growth rate remains [32]. To our knowledge, although the effects of individual growth rates on TDFs have been demonstrated in fish species, similar evaluations have not been conducted in endotherms with higher basal metabolic rates, like birds [33, 34], but see [35]. Because variation in tissue isotopic values related to environmental factors and dietary composition (isotopic values and source of macronutrients) is minimized in our captive setting, the broiler chicks are an ideal system to test how variation in growth and other organism-dependent factors, such as tissue type, affect TDFs and how accurately BSIMMs allocate individual birds to assigned dietary groups when model inputs vary.

We anticipated that both individual growth rate and tissue type would be important predictors of *Δ*^13^C values, with *Δ*^13^C decreasing as growth rate increases and routing of dietary constituents (especially amino acids) to tissues is prioritized over the production of energy (the “anabolism hypothesis” [36, 37]. We also expected the growth rate effect to be tissue dependent, being more evident in liver, a tissue that is vital for bodily function and has more rapid turnover, relative to feathers, which are metabolically inert [38]. For *Δ*^15^N, we expected that tissue type would have a similar effect as *Δ*^13^C (liver having lower values compared to feathers), but that effects of metabolic routing would be reduced, resulting in a lesser effect of growth on *Δ*^15^N, relative to *Δ*^13^C [39]. Model outputs from BSIMMs should provide the most accurate assessments of assimilated diets when observed variation in TDFs (i.e., diet-specific *Δ*^13^C and *Δ*^15^N values) are applied [19, 40]. In terms of uncertainty estimates, we presume that birds in our study could not preferentially consume dietary components, so that process error (i.e., individual specialization) will have a minimal influence on isotopic variation of tissues. We therefore predict that models specifying a residual error-only formulation will be most supported by the data [15, 16].

## Methods

### Diet composition and experimental design

Using commercially available feed ingredients, we designed two isotopically distinct, nutritionally balanced diets to meet or exceed nutrient specifications for as-hatched Ross-308 broiler chickens [41]. Detailed summaries of dietary constituents are available as Supporting Information (S1 Table). The two primary diet formulations (diet 1 and diet 2) were manufactured in mash form, with two secondary formulations based on mixtures: diet 3 (67% diet 1; 33% diet 2) and diet 4 (33% diet 1; 67% diet 2). All four diets were then pelleted (< 80°C in a conditioner) and crumbled, with each dietary treatment designated as an experimental group. Day of hatch, mixed-sex Ross-308 broiler chicks were purchased from a commercial hatchery (Sofina Foods Inc., Wynyard, Saskatchewan, Canada), weighed, individually banded, and randomly assigned to one of 24 cages (six replicate cages per group). Each experimental unit (i.e., cage) contained four chicks, and to replace any birds that died during the first week of the trial, we also maintained extra chicks for each group (n = 10 per group) in separate cages.

Housing procedures followed standard production guidelines for caged broiler chickens, and we obtained written consent and approval for all protocols from the Animal Care Committee (Animal Research Ethics Board), University of Saskatchewan, on behalf of the Canadian Council on Animal Care (Protocol 19940248, Poultry Centre File Number CTR 1702). We placed individual birds in the top compartments (51 cm W x 51 cm L x 46 cm H; 650 cm^2^ per bird) of two-tier battery cages and gave birds *ad libitum* access to feed (front-mounted feed trough) and water (nipple, automatic, adjustable height). Extra feed was provided in supplemental trays for the first four days, and water was provided in ice cube trays for the first week. Room temperature was gradually reduced (3°C weekly) from 34°C to 22°C. The light:dark cycle began at 23:1 for day-old chicks and was gradually adjusted to 19:5 by day six, where it remained for the remainder of the trial (total of 29 days). The light period included 15-minute dawn and dusk periods, and light intensity at the feeder was 20 lux. To eliminate the possibility of dietary contamination across groups, chicks were placed only in the top tier of cages, and an empty cage was left between experimental replicates.

To index feed intake and growth rates, changes in food mass were estimated over a 24-hour period throughout the trial and on days one, 14, and 29, remaining chicks (n = 93; one bird censored due to a missing mass value at day 29) were weighed on a top-loading balance (± 1 g). Dietary samples from each group were collected throughout the trial (weekly) to test isotope values of feed. After 29 days, we euthanized all birds via cervical dislocation. We immediately excised the entire liver, collected flank-breast feathers, and sexed birds via visual confirmation of testes or ovaries. Liver and feather samples were stored in sterile polyethylene bags at -80°C (liver) or in paper envelopes at -18°C (feathers).

### Stable isotope analyses

Feather samples were rinsed with a 2:1 chloroform-methanol solution, allowed to air dry for 24 hours, and finely cut into pieces (calamus removed); liver samples were freeze-dried and ground to a homogenous powder; and diet samples were finely powdered in a homogenizer. To remove lipids, liver and diet samples were rinsed with a 2:1 chloroform-methanol solution and dried to constant mass. All tissue and diet samples were packed (approximately 1 mg) into tin capsules and combusted in a Costech (Milan, Italy) ECS4010 elemental analyzer interfaced with a Delta V continuous-flow isotope-ratio mass spectrometer at the Department of Soil Science, University of Saskatchewan. By convention, isotope values are reported in delta notation as *δ*^13^C and *δ*^15^N according to:

$$\delta X=\left(\frac{R_{sample}}{R_{std}}-1\right)\%$$

where X = ^13^C or ^15^N, R = ^13^C:^12^C or ^15^N:^14^N, and international standards (std) are Vienna PeeDee Belemnite (VPDB) for ^13^C and atmospheric N_2_ (AIR) for ^15^N, and where measured delta values were converted to international isotope reference scales using two-point linear normalization [42].

Two secondary isotopic reference materials (egg albumen and lyophilized bowhead whale baleen) that were expected to span the range of experimental values were selected by the analytical lab, as standards to allow estimation of measurement precision [43]. Standards–provided by the lab (in the same mass range as test samples)–were calibrated by running them against USGS 40 and USGS 41a L-glutamic acids, which have internationally accepted *δ*^13^C and *δ*^15^N values [44, 45]. For further quality assurance / quality control, standards were processed before and between every five tissue samples within each analytical run (for albumen) and at the start, midpoint, and end of each run (for baleen). We used replicate standard measurements within a run to estimate analytical precision: mean *δ*^13^C (± 1 standard deviation, SD) within runs = –22.4 ‰ ± 0.0 ‰, and mean *δ*^15^N (± 1 SD) within runs = 6.8 ‰ ± 0.1 ‰ for egg albumen; mean *δ*^13^C (± 1 SD) within runs = –18.5‰ ± 0.1%, and mean *δ*^15^N (± 1 SD) within runs = 14.8 ‰ ± 0.2%, for bowhead whale baleen. Measurement error estimates (± 1 SD) were ± 0.1 ‰ for *δ*^13^C and ± 0.3 ‰ for *δ*^15^N, based on measurements of laboratory standards across multiple runs [43].

### Statistical approach

To evaluate the relative influence of organism-related factors (growth rate and tissue type) on TDFs, analyses were implemented in SAS^®^ Version 9.4 (SAS^®^ Inc., Cary, NC). We calculated *Δ*^13^C as *δ*^13^C_Tissue_− *δ*^13^C_Diet_ and *Δ*^15^N as *δ*^15^N_Tissue_− *δ*^15^N_Diet_ for both liver and feathers, estimated growth parameters for each individual, based on three-parameter Gompertz growth curves [46, 47], and used general linear mixed models for our analyses. These models allowed us to account for dependence among birds at the cage level (random effects), while evaluating the specific and separate influences of our predictor variables of interest [48]. For each TDF, model covariance structure was determined based on data structure and convergence criteria [49]. Marginal log-likelihood values for each model were computed using restricted maximum likelihood methods, and a Kenward-Roger correction was applied in computing denominator degrees of freedom (PROC MIXED) [50]. After developing an *a priori* set of candidate models (one each for *Δ*^13^C and *Δ*^15^N), we compared models based on an information-theoretic approach, with models ranked according to 2nd-order Akaike’s Information Criterion corrected for sample size (AIC_c_) and relative likelihoods for each model estimated by Akaike weights (*w*_i_) [51].

Key predictor variables (fixed effects) in our models included individual growth rate based on mass (Mass_B_), tissue type, and their interaction (Mass_B_*Tissue). To account for additional factors known to affect TDFs, we also included diet type (Group) and sex in our model sets [40, 52] and evaluated potential interactions between diet and tissue (Group*Tissue) and sex and tissue (Sex*Tissue). Inference concerning fixed effects was based on precision (85% confidence interval, CI) of the regression coefficient (β), as this interval is more compatible with the information-theoretic approach. When wide confidence intervals indicated imprecise slope estimates, we considered respective covariates as uninformative [53].

To compare the effects of different TDFs and error structures on stable isotope dietary inference and test how accurately BSIMMs estimated known proportions of the diets that were consumed, we used hierarchical BSIMMs, implemented in the open-source R package, MixSIAR Version 3.1.10 [54]. Within MixSIAR, each model was first run under test settings to confirm models were correctly specified–cage as a random effect, nested within the fixed effect of group (Cage(Group)). For final analyses, Gibbs sampling parameters were set to 3 parallel chains of 300,000 vectors (burn-in = 200,000, thinning interval = 100). To determine whether mixing models converged, we examined trace plots for relatively constant mean and variance, as well as for small fluctuations in the estimated parameters–results from the Gelman-Rubin diagnostic test were also considered [54]. TDFs were specified as diet-specific (mean ± standard deviation)(Diet 1: *Δ*^13^C = 0.7 ± 0.2, *Δ*^15^N = 0.7 ± 0.2; Diet 2: *Δ*^13^C = 2.3 ± 0.2, *Δ*^15^N = 1.2 ± 0.4) or combined (*Δ*^13^C = 1.4 ± 0.8, *Δ*^15^N = 0.9 ± 0.3). Different specifications of BSIMMs that converged were ranked using the Deviance Information Criterion, DIC [55], and to estimate the relative accuracy of model outputs across groups, we compared the actual diet proportions to the model-based 95% credible intervals–when intervals included the known proportion, we considered the output accurate.

## Results

### Diet composition and chicken growth

**Fig 1:**
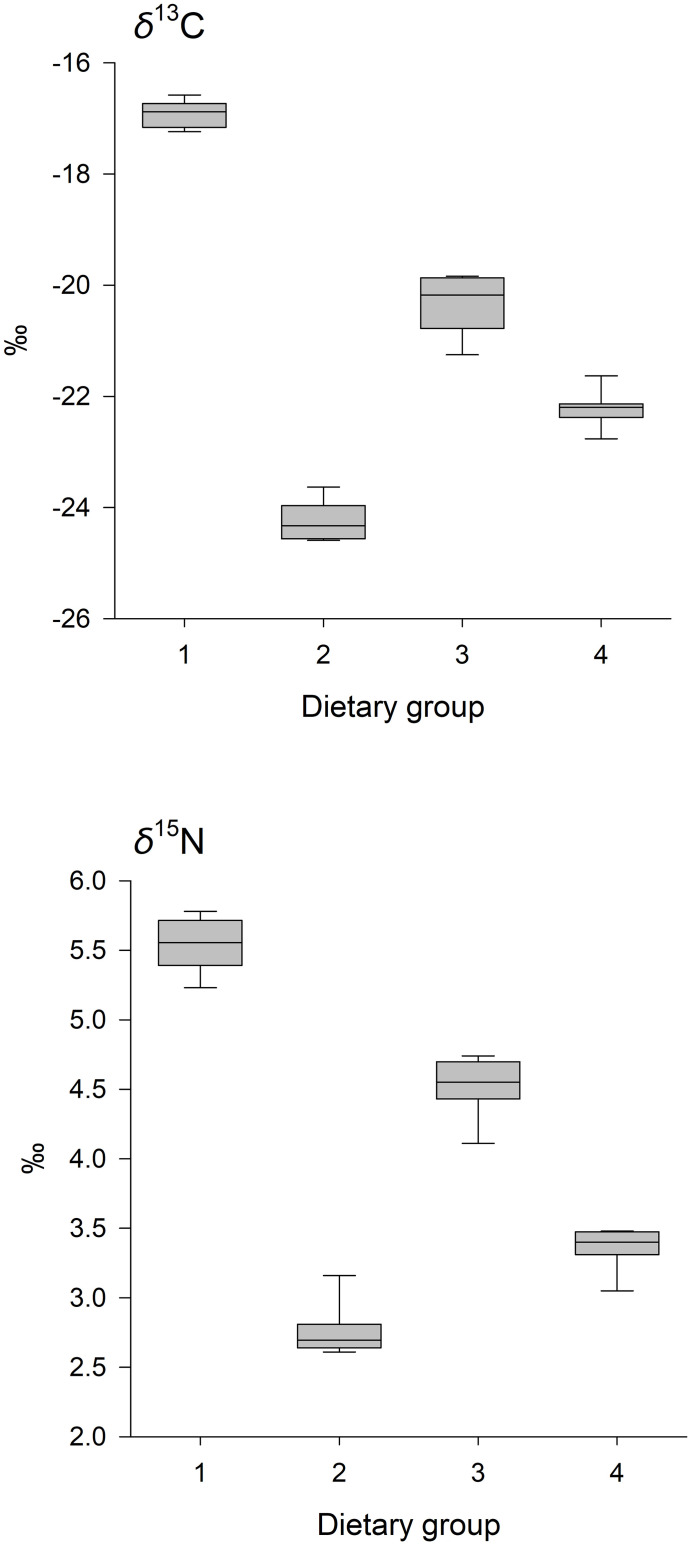
Dietary *δ*^13^C (upper panel) and *δ*^15^N (lower panel) by experimental dietary group. Isotope values differed among diets fed to growing Ross-308 strain chicks under controlled environmental conditions. Shown are the median (horizontal line within shaded boxes), lower and upper quantiles (lower and upper edge of box, respectively) and range (vertical line) for each dietary group (n = 8 samples per group).

Feed isotopic values (*δ*^13^C, *δ*^15^N) were significantly different among the experimental diet groups (MANOVA, Wilks’ λ = 0.007, F_6, 54_ = 97.06, p < 0.0001); as expected, our formulations resulted in a high *δ*^13^C / *δ*^15^N diet (Group 1), a low *δ*^13^C / *δ*^15^N diet (Group 2) and 2 intermediates (Groups 3 and 4) (Fig 1).

On day one, chick body mass averaged 47.1 g (± 1.0 g standard error, SE), and no effects of sex or dietary grouping were detected. Group 3 had three more females than males and Group 1 had six more males than females, but sex ratio did not differ among groups (G-test, G_3_ = 1.91, p = 0.59); overall, there were 46 females and 48 males (Table 1). By day 29, after accounting for cage effects, additional effects of sex (p < 0.0001) and diet group (p = 0.01) on body mass were detected, with no interaction. Across all birds, coefficients for daily rate of mass gain (Mass_B_) ranged from 0.0403 (a bird in Group 3) to 0.0907 (a bird in Group 3), solving the Gompertz equations for days 15 and 16, estimated values were equivalent to 34 to 83 g of body mass gained per day. Although we detected no effects of sex or diet group (or their interaction) on Mass_B_, by the end of the diet trial, female chickens (least squares mean = 1621 g ± 28 SE) averaged ~144 g less than males (1765 g ± 28 SE). Adjusting for sex effects, least square mean body masses (SE) of chickens were 1732 g (46), 1737 g (46), 1539 g (47), 1736 g (46) for diet Groups 1–4, respectively.

**Table 1:** Descriptors of growth for Ross-308 broiler chicks.

Dietary Group	Sex	n	Mass_29_ (g)	B	M_*m*_ (g)	t*
**1**	Female	9	1655 (33)	0.0634 (0.0015)	3510 (181)	24 (1)
	Male	15	1807 (28)	0.0639 (0.0018)	3915 (218)	25 (1)
**2**	Female	12	1665 (38)	0.0652 (0.0013)	3362 (157)	23 (1)
	Male	12	1809 (56)	0.0626 (0.0029)	4289 (414)	26 (1)
**3**	Female	13	1426 (66)	0.0576 (0.0022)	3382 (149)	27 (1)
	Male	9	1657 (76)	0.0625 (0.0040)	3800 (344)	25 (2)
**4**	Female	12	1704 (26)	0.0626 (0.0017)	3704 (199)	25 (1)
	Male	11	1819 (41)	0.0611 (0.0017)	4156 (175)	26 (1)

Mean (standard error) for each dietary group were estimated using a Gompertz three-parameter equation, M_*t*_ = M_*m*_ exp(–exp(-B(*t*–t*))), where M_*t*_ = mass at time *t*, M_*m*_ = estimated mass at maturity, B = coefficient for daily rate of mass gain, and t* = day on which mass gain is maximized.

### Stable isotope values & factors affecting TDFs

**Fig 2:**
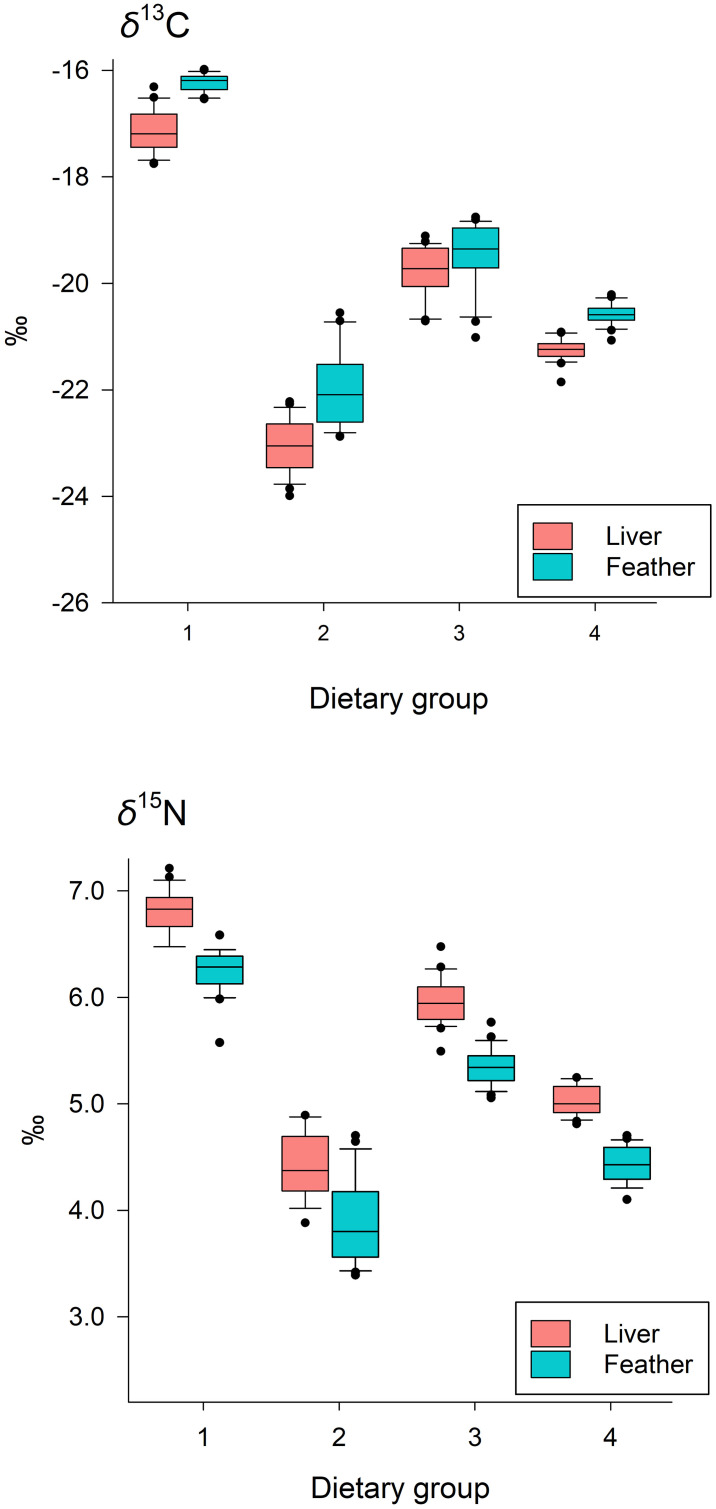
Tissue *δ*^13^C (upper panel) and *δ*^15^N (lower panel) for liver and feather. Values varied across dietary groups. Shown are the median (horizontal line within boxes), lower and upper quantiles (lower and upper edge of box, respectively) and range (vertical line) for each dietary group, with values above or below the interquartile range (upper quartile–lower quartile) indicated by closed circles.

**Fig 3:**
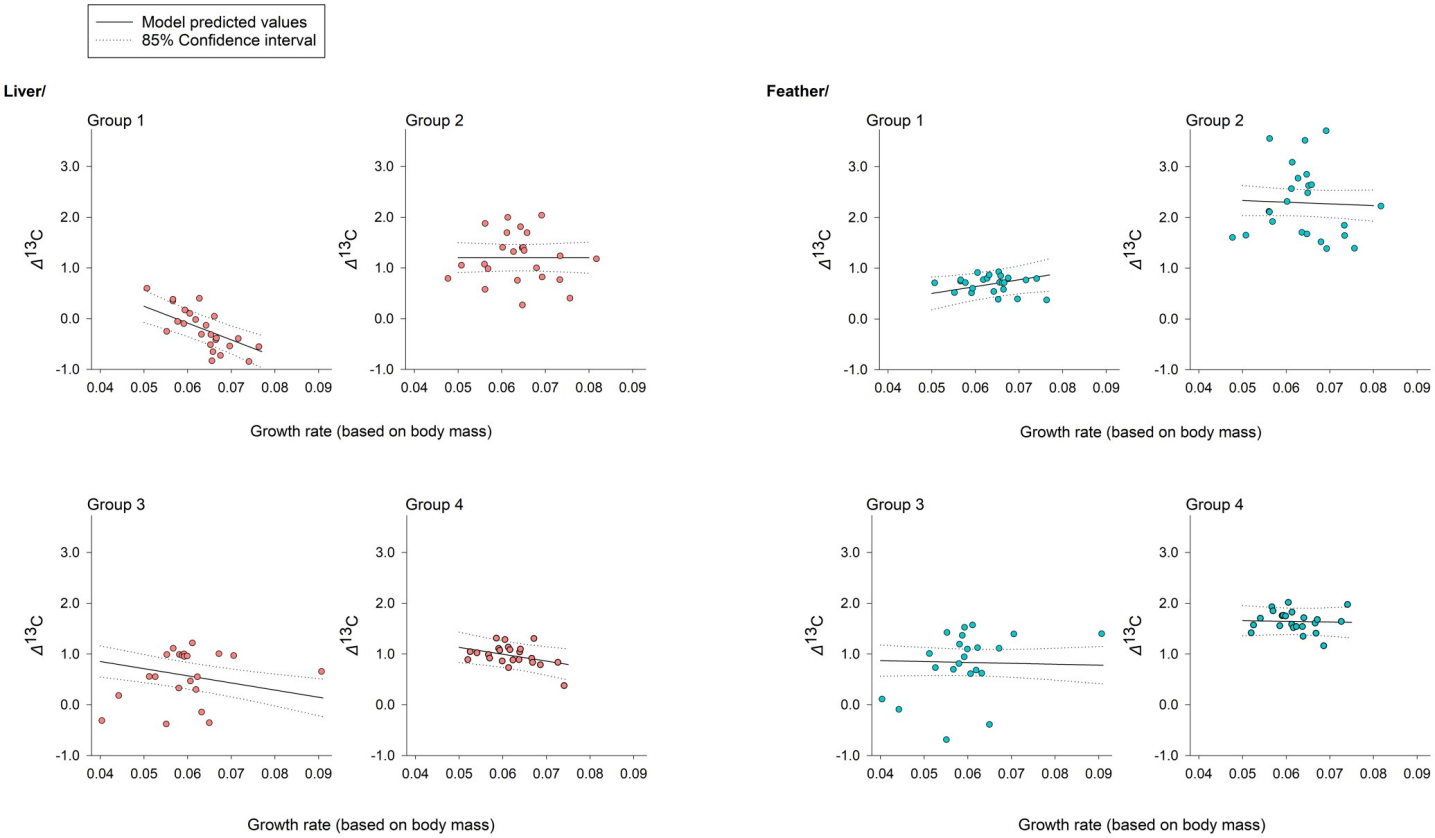
*Δ*^13^C in captive broiler chicks was affected by individual growth rate. The effect varied across dietary groups and between tissue types. All plots show model-based estimates (± 85% confidence intervals) as indicated in the legend. Raw data (circle symbols) are plotted for visual comparison.

**Fig 4:**
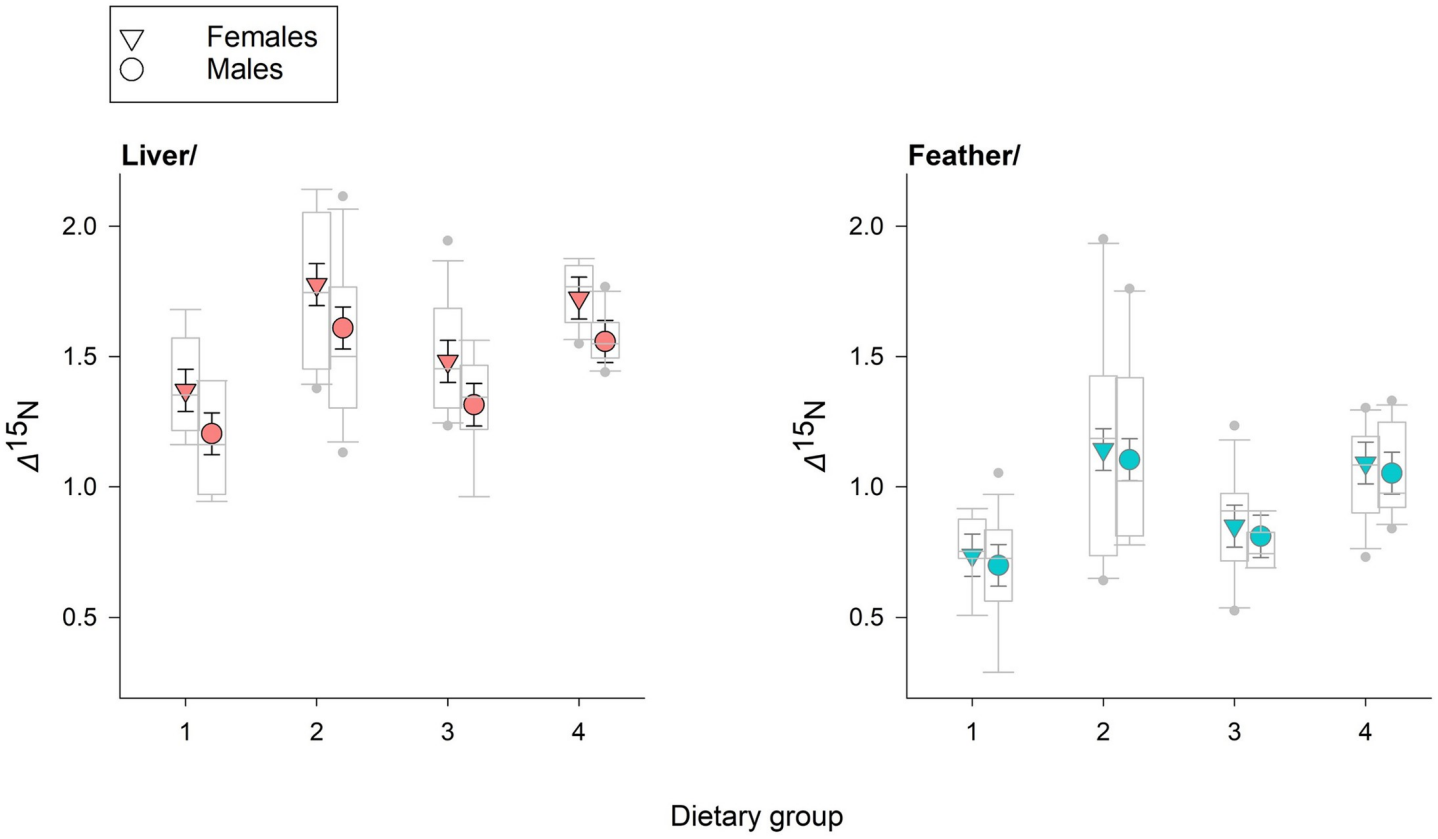
Dietary group, sex, and tissue type influenced *Δ*^15^N in captive broiler chicks. For liver, *Δ*^15^N was higher in females (triangles), but for feathers, values were similar between females and males (circles). All plots show model-based estimates (± 1 standard error). Raw data (grey boxplots) are plotted for visual comparison.

For both liver and feather tissue, *δ*^13^C and *δ* ^15^N values differed among dietary groups, reflecting patterns in feed isotope values; compared to feather, *δ*^13^C was generally more negative for liver, with the opposite pattern for *δ*^15^N (Fig 2). Similarly, as expected, *Δ*^13^C was affected by dietary group, tissue type, and their interaction, but models including an effect of sex on *Δ*^13^C were not supported. Consistent with our predictions under the anabolism hypothesis, we detected a negative effect of Mass_B_ on *Δ*^13^C, for liver tissue only (β_MassB*Feather_ = 13.7, 85% CI = 7.0 to 20.5), with some minor differences among groups (Table 2, Fig 3). For *Δ*^15^N, the most-supported model included effects of dietary group, sex, and tissue type, as well as an interaction between sex and tissue type (*w*_i_ = 0.63; β_Female*Feather_ = -1.30, 85% CI = -0.19 to -0.06), although a model without the interaction also received moderate support (*w*_i_ = 0.36, Table 3). The sex effect (females having higher *Δ*^15^N relative to males) was observed only for liver, and contrary to our expectation, in general, *Δ*^15^N values were greater for liver than feather (Fig 4).

**Table 2:** Model ranking for *Δ*^13^C.

Model Structure	K	-2logL	ΔAIC_c_	*w*_i_
Group*Tissue + Mass_B_*Tissue	12	64.7	0.00	1.0
Group*Tissue + Mass_B_	11	78.0	11.01	0.0
Group + Tissue + Mass_B_	8	132.1	58.41	0.0
Group + Tissue	7	139.4	63.53	0.0
Intercept	3	329.4	245.03	0.0

Results are consistent with tissue-dependent effects of individual growth rate (Mass_B_) on *Δ*^13^C. For all models, cage nested within group is included as a random effect. -2logL = Deviance, *w*_i_ = Akaike weight. K = number of parameters estimated. The + between variables indicates an additive effect, the * denotes interaction; where interactions are listed, main effects were also included.

**Table 3:** Model ranking for *Δ*^15^N.

Model Structure	K	-2logL	ΔAIC_c_	*w*_i_
Group + Sex*Tissue	9	-84.5	0.00	0.63
Group + Tissue + Sex	8	-81.2	1.09	0.36
Group + Tissue	7	-72.3	7.81	0.01
Intercept	3	170.0	241.61	0.00

The model most supported by our data identified that sex affected *Δ*^15^N for liver tissue only. Cage nested within group is included as a random effect. -2logL = Deviance, *w*_i_ = Akaike weight. K = number of parameters estimated. The + between variables indicates an additive effect, the * denotes interaction; where interactions are listed, main effects were also included.

### Influences of TDFs & error structure on performance of BSIMMs

**Fig 5:**
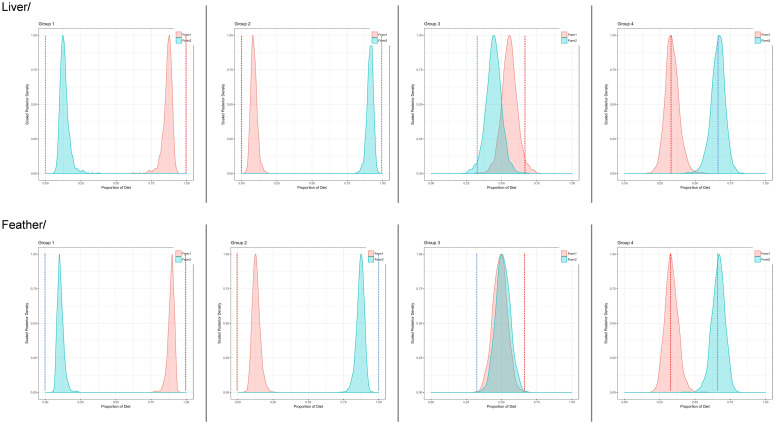
Estimated contributions (Bayesian credible intervals for scaled posterior densities) of each formula (= ‘form’, as legend), for both liver and feather. Estimates based on top-ranked Bayesian stable isotope mixing models were similar to known dietary contributions (indicated by vertical dotted lines), with some variation in accuracy related to dietary group and tissue type.

For liver tissue, our top-ranked BSIMM (based on DIC) showed that combining the variance in TDFs across diet types and applying a multiplicative error structure would improve model accuracy compared to the residual error-only model (ΔDIC = 6.6; Table 4). Models with diet-specific TDF values for *Δ*^13^C and *Δ*^15^N either did not converge (multiplicative error) or were poorly supported by the data (residual error). Results for feather models were similar in that increasing variance estimates for TDF values–by combining diet-specific values–and using a multiplicative error structure resulted in the most-supported model, although there was some indication that a residual error-only model (with combined TDF variance) performed well (ΔDIC = 2.5; Table 5). Outputs from our top-ranked BSIMMs were generally very similar to known dietary contributions, with some variation in accuracy, depending on group and tissue type. For liver, estimated dietary proportions based on 95% credible intervals were accurate (i.e., included known proportions) for dietary groups 3 and 4, whereas for feather–best model outputs were accurate for group 4 only (Tables 4 and 5). Elsewise, credible intervals generally were within 10% of known proportions (Fig 5).

**Table 4:** Bayesian stable isotope mixing model outputs for liver tissue.

TDF-Variance	Error term	Dietary group 1		Dietary group 2		Dietary group 3		Dietary group 4		DIC
		Formula 1 (1)^a^	Formula 2 (0)	Formula 1 (0)	Formula 2 (1)	Formula 1 (0.67)	Formula 2 (0.33)	Formula 1 (0.33)	Formula 2 (0.67)	
Combined	P*R	0.87, 0.78–0.91	0.13, 0.09–0.22	0.09, 0.05–0.14	0.91, 0.86–0.95	0.56, 0.45–0.67	0.44, 0.33–0.55	0.33, 0.25–0.44	0.67, 0.56–0.76	38.8
Combined	R	0.87, 0.80–0.91	0.13, 0.09–0.20	0.08, 0.05–0.13	0.92, 0.87–0.95	0.55, 0.45–0.66	0.45, 0.35–0.56	0.34, 0.24–0.44	0.66, 0.56–0.76	45.4
Diet specific	P*R	Did not converge								
Diet specific	R	0.87, 0.49–0.96	0.13, 0.04–0.51	0.06, 0.02–0.26	0.94, 0.74–0.98	0.61, 0.30–0.87	0.39, 0.13–0.70	0.39, 0.16–0.76	0.61, 0.24–0.84	117.4

Using trophic discrimination factors with increased variance (i.e., combined across diet types) and an error structure that allows for combinations of process error (i.e., individual specialization and sampling error) as well as residual error, yielded the best-approximating Bayesian stable isotope mixing model. Model specifications are identified in the first two columns, known dietary proportions are shown for each experimental group^a^, as well as estimated dietary contributions from each model (median, 95% credible interval). Where the credible interval includes the known proportion (i.e., model output is accurate), cells are shaded green. P = process error, R = residual error. DIC = Deviance Information Criterion.

^a^ Bolded values in parentheses indicate known dietary proportions.

**Table 5:** Bayesian stable isotope mixing model outputs for feather tissue.

TDF-Variance	Error term	Dietary group 1		Dietary group 2		Dietary group 3		Dietary group 4		DIC
		Formula 1 (1)^a^	Formula 2 (0)	Formula 1 (0)	Formula 2 (1)	Formula 1 (0.67)	Formula 2 (0.33)	Formula 1 (0.33)	Formula 2 (0.67)	
Combined	P*R	0.90, 0.84–0.93	0.10, 0.07–0.16	0.13, 0.08–0.19	0.87, 0.81–0.92	0.49, 0.39–0.59	0.51, 0.41–0.61	0.33, 0.25–0.43	0.67, 0.57–0.75	66.8
Combined	R	0.89, 0.81–0.93	0.11, 0.07–0.19	0.12, 0.07–0.18	0.88, 0.82–0.93	0.51, 0.40–0.62	0.49, 0.38–0.60	0.33, 0.24–0.45	0.67, 0.55–0.76	69.3
Diet specific	P*R	0.84, 0.19–0.97	0.16, 0.04–0.81	0.10, 0.02–0.54	0.90, 0.49–0.98	0.56, 0.11–0.85	0.45, 0.15–0.89	0.39, 0.11–0.82	0.61, 0.18–0.89	91.7
Diet specific	R	0.83, 0.28–0.95	0.17, 0.05–0.72	0.11, 0.02–0.55	0.89, 0.45–0.98	0.60, 0.22–0.89	0.40, 0.11–0.79	0.40, 0.10–0.79	0.60, 0.21–0.90	108.8

Using trophic discrimination factors with increased variance (i.e., combined across diet types) and an error structure that allows for combinations of process error (i.e., individual specialization and sampling error) as well as residual error, yielded the best-approximating Bayesian stable isotope mixing model. Model specifications are identified in the first two columns, known dietary proportions are shown for each experimental group^a^, as well as estimated dietary contributions from each model (median, 95% credible interval). Where the credible interval includes the known proportion (i.e., model output is accurate), cells are shaded green. P = process error, R = residual error. DIC = Deviance Information Criterion.

^a^ Bolded values in parentheses indicate known dietary proportions.

## Discussion

Using captive, domestic birds being fed specifically formulated, isotopically distinct diets, we have directly measured how several factors, including diet, tissue type, sex, and individual growth rates, affect trophic discrimination factors and, further, how variation in the specifications of Bayesian stable isotope mixing models affect the accuracy of their outputs. Although TDFs varied considerably across dietary groups and tissue types (as expected), our work highlights two other factors that influence TDFs in birds–sex and individual growth rate. Importantly, we also show that adequate estimates of uncertainty in BSIMMs improve performance of these models, and that when variation in the isotope values of animals due to environment and food resources is controlled (i.e., secondary mechanistic factors; 18), BSIMM-derived estimates of consumed diet can closely reflect known proportions.

### Factors influencing trophic discrimination factors

As expected, our analyses indicated a negative relationship between individual growth rate and *Δ*^13^C –for liver tissue only. Although the relationship between growth rates and TDFs has rarely been investigated directly, this finding is generally aligned with other studies of vertebrates that suggest an important role for metabolic processes in determining trophic discrimination factors [56–58]. For example, faster growing juvenile fish tend to have higher metabolic rates, relative to slower-growing conspecifics, which in turn is linked to decreases in *Δ*^13^C [36, 59]. It is unclear if adaptations to endothermy would result in similar relationships for birds. Intraspecific variation in percentage mass change did not affect turnover rates of ^13^C in dunlin (*Calidris alpina*), and *Δ*^13^C did not differ predictably between slower and faster-growing captive American crows (*Corvus brachyrhynchos*) [38, 60]. Yet *Δ*^13^C in domestic chickens ranges from approximately -4‰ to 2‰, and observed variation among captive-raised *Caipirinha* broilers suggests a growth effect in this species [40, 61], as do the lower TDFs for younger age classes that have been observed in other species [62]. Our results thus add to a growing body of evidence that differences in physiology among individuals have an important influence on TDFs, which we speculate is linked to increased routing of dietary protein to body tissues during rapid growth (i.e., anabolism). That we observed an inverse relationship between growth rate and *Δ*^13^C only in liver, a highly metabolically active tissue (relative to feathers), is consistent with this idea [63, 64]. However, differences in the specific amino acid composition of livers and feather (i.e., keratin) may be an additional factor affecting differences in fractionation values between tissue types and the interaction with growth rate [65, 66].

For *Δ*^15^N, our finding that diet and tissue type are reliable predictors of fractionation values is consistent with expectations, which were based on other studies that consistently highlight the importance of these factors, especially dietary composition [21, 22, 67]. The sex dependence of the tissue effect on *Δ*^15^N, however, was not anticipated. In liver only, fractionation values for nitrogen were marginally higher for female chicks than for males. Although few studies have specifically evaluated sex effects on TDFs, higher *Δ*^15^N in females have previously been noted in some mammalian species [68, 69]. Although sex-based variation in growth is a proposed mechanistic explanation in both cases, this hypothesis is unsupported by our study, where there was no evidence of a growth rate effect on *Δ*^15^N. Instead, physiological differences related to oogenesis may play an important role. If female broiler chicks increase routing of nitrogen to liver for production of eggs–even prior to sexual maturity–enrichment of ^15^N would be expected [70].

We did not design our feeding trial to specifically evaluate the influence of dietary composition (isotopic values and sources of macronutrients) on trophic discrimination factors for carbon or nitrogen, but our findings that dietary group affected both *Δ*^13^C and *Δ*^15^N were not unexpected. Accounting for other factors, in our study, *Δ*^13^C and *Δ*^15^N were generally highest for dietary groups 2 and 4 (Figs 3 and 4), which had reduced *δ*^13^C values (Fig 1), a greater percentage of C_3_-based carbon (i.e., wheat and soy), and less fish meal, relative to Groups 1 and 3. Although evidence of an influence of *δ*^13^C values on *Δ*^13^C in birds is lacking [71], origin of carbon sources (i.e., C_3_-based, C_4_-based, marine-based, or a mixture) and dietary protein content can explain substantial variation in *Δ*^13^C for avian species [39, 40]. Given similar estimated protein content across the different diets in our study, our data support a carbon source hypothesis, but we note that differences in digestibility of different protein sources may also be important, not only for *Δ*^13^C, but also for *Δ*^15^N [67, 72]. In particular, for *Δ*^15^N, our results are consistent with an important influence of protein quality; our diet groups with higher levels of easily-digestible fish protein also had lower *Δ*^15^N values. Additional studies–especially those using specifically formulated food sources and compound-specific isotope analysis of amino acids–will provide improved insight about how diet composition affects *Δ*^13^C [73, 74].

### Performance of Bayesian stable isotope mixing models

Varying TDF estimates and error structures in our BSIMM formulations affected performance of the models (based on DIC and estimated dietary contributions), with variance in TDFs having the greater effect, both for liver and feather tissue. Although we anticipated that model performance would be improved by specifying diet-specific *Δ*^13^C and *Δ*^15^N values–we observed the opposite. When we included estimates of TDFs that were combined across dietary sources, model performance and accuracy of diet assignments were improved, relative to models with diet-specific estimates. Although this is in apparent contrast to the idea that diet-specific estimates of TDFs are more valuable for isotopic dietary assignments than literature-based values [5, 19], our findings align with work suggesting that larger uncertainties in TDFs can improve fit and accuracy of BSIMMs [24, 75].

Regarding the accuracy of BSIMM outputs under best-case conditions, we note that under both TDF specifications, relative dietary contributions estimated by the Bayesian credible intervals were similar to known proportions (Tables 4 and 5), being within about 10% of the known diet (or better) in all cases we looked at. This is encouraging for use of BSIMMs to identify diets in field studies, albeit not perfect. Importantly, our results, particularly for dietary groups 3 and 4, suggest that dominant dietary components would be clearly identified by BSIMM outputs, even though accuracy of estimates might vary. Ecologists can therefore expect predicted diets based on these models will give solid insights into the dominant assimilated dietary components, at least in food webs with isotopically distinct dietary options. We recommend, however, that results not be considered as indicative of strictly true diets, as revealed in our “moment of truth”.

Because our results indicate that accuracy, more than inference, is affected by TDF variance estimates when isotopic differences between source proportions are high, we recommend that future studies should consistently consider overall sensitivities of BSIMM outputs to TDF variance, particularly because assuming single, fixed TDFs can artificially reduce estimates of sampling error and the importance of ecological processes such as individual specialization [16]. That models with process-related error terms (for both tissue types) were more supported by our data than residual error only models is consistent with the idea that variability in consumer isotope values due to chance–as well as due to distinct dietary choices of individual animals–are important elements of mixing models. This was an unexpected finding for our captive setting, where we expected that the relatively homogenous nature of the pelleted feed would prevent individuals from preferentially consuming one formula (i.e., source) over another. We also assumed that by using the same lipid extraction methods for all diet and liver samples (feathers do not contain lipids), possible effects of lipid extraction on BSIMM outputs would be negligible [76, 77]; indeed, model-predicted dietary assignments for lipid-rich liver and lipid-free feather tissues were similar (Fig 5).

Although the importance of process-related error in our models implies that source selectivity among individual consumers may be underestimated in many natural settings, we caution that factors leading to individual specialization are likely dependent on variation in an individual’s state, as well as competitive pressure for resources [78, 79]. We suggest that additional studies assessing intraspecific dietary variation are needed to fully understand the extent of this phenomenon and the conditions under which it is expected to arise. We also further echo the suggestions of others that understanding the underlying ecological assumptions of different BSIMM formulations, particularly error structures, is key to improving inference in dietary studies [13, 80, 81].

## Supporting information

S1 Table Commercially available feed ingredients were used to formulate experimental diets. Percentages of each constituent are shown.
(PDF)

## References

[1] Pinzone M, Damseaux F, Michel LN, Das K. Stable isotope ratios of carbon, nitrogen and sulphur and mercury concentrations as descriptors of trophic ecology and contamination sources of Mediterranean whales. Chemosphere. 2019;237:124448. doi: 10.1016/j.chemosphere.2019.124448 31398606

[2] Bonin M, Dussault C, Taillon J, Lecomte N, Côté SD. Combining stable isotopes, morphological, and molecular analyses to reconstruct the diet of free-ranging consumers. Ecology and Evolution. 2020;10(13):6664–76. doi: 10.1002/ece3.6397 32724540 PMC7381590

[3] Gurney K, Clark R, Slattery S, Ross L. Connecting the trophic dots: responses of an aquatic bird species to variable abundance of macroinvertebrates in northern boreal wetlands. Hydrobiologia. 2017;785(1):1–17.

[4] Potapov AM, Tiunov AV, Scheu S. Uncovering trophic positions and food resources of soil animals using bulk natural stable isotope composition. Biological Reviews. 2019;94(1):37–59. doi: 10.1111/brv.12434 29920907

[5] Johnson DL, Henderson MT, Anderson DL, Booms TL, Williams CT. Bayesian stable isotope mixing models effectively characterize the diet of an Arctic raptor. Journal of Animal Ecology. 2020;89(12):2972–85. doi: 10.1111/1365-2656.13361 33020919

[6] Cherel Y, Jaquemet S, Maglio A, Jaeger A. Differences in δ 13 C and δ 15 N values between feathers and blood of seabird chicks: implications for non-invasive isotopic investigations. Marine Biology. 2014;161:229–37.

[7] Steenweg RJ, Crossin GT, Kyser TK, Merkel FR, Gilchrist HG, Hennin HL, et al. Stable isotopes can be used to infer the overwintering locations of prebreeding marine birds in the Canadian Arctic. Ecology and Evolution. 2017;7(21):8742–52. doi: 10.1002/ece3.3410 29177032 PMC5689493

[8] Graves SD, Liber K, Palace V, Hecker M, Doig LE, Janz DM. Trophic dynamics of selenium in a boreal lake food web. Environmental Pollution. 2021;280:116956. doi: 10.1016/j.envpol.2021.116956 33799129

[9] Phillips DL, Inger R, Bearhop S, Jackson AL, Moore JW, Parnell AC, et al. Best practices for use of stable isotope mixing models in food-web studies. Canadian Journal of Zoology. 2014;92(10):823–35.

[10] Fry B. Stable isotope ecology: Springer; 2006. 308 p.

[11] Wefing AM, Casacuberta N, Christl M, Gruber N, Smith JN. Circulation timescales of Atlantic Water in the Arctic Ocean determined from anthropogenic radionuclides. Ocean Sci. 2021;17(1):111–29.

[12] Smith HG, Karam DS, Lennard AT. Evaluating tracer selection for catchment sediment fingerprinting. Journal of Soils and Sediments. 2018;18(9):3005–19.

[13] Stock BC, Jackson AL, Ward EJ, Parnell AC, Phillips DL, Semmens BX. Analyzing mixing systems using a new generation of Bayesian tracer mixing models. PeerJ. 2018;6(e5096):e5096. doi: 10.7717/peerj.5096 29942712 PMC6015753

[14] Liu J, Han G. Tracing Riverine particulate black carbon sources in Xijiang river Basin: insight from stable isotopic composition and Bayesian mixing model. Water Research. 2021;194:116932. doi: 10.1016/j.watres.2021.116932 33640754

[15] Parnell AC, Inger R, Bearhop S, Jackson AL. Source partitioning using stable isotopes: coping with too much variation. PLoS One. 2010;5(3):Article. doi: 10.1371/journal.pone.0009672 20300637 PMC2837382

[16] Stock BC, Semmens BX. Unifying error structures in commonly used biotracer mixing models. Ecology. 2016;97(10):2562–9. doi: 10.1002/ecy.1517 27859126

[17] Cooper RJ, Krueger T, Hiscock KM, Rawlins BG. Sensitivity of fluvial sediment source apportionment to mixing model assumptions: AB ayesian model comparison. Water Resources Research. 2014;50(11):9031–47. doi: 10.1002/2014WR016194 26612962 PMC4650832

[18] Boecklen WJ, Yarnes CT, Cook BA, James AC. On the use of stable isotopes in trophic ecology. Annual review of ecology, evolution, and systematics. 2011;42:411–40.

[19] Kadye WT, Redelinghuys S, Parnell AC, Booth AJ. Exploring source differences on diet-tissue discrimination factors in the analysis of stable isotope mixing models. Scientific reports. 2020;10(1):15816. doi: 10.1038/s41598-020-73019-x 32978550 PMC7519091

[20] Bond AL, Diamond AW. Recent Bayesian stable-isotope mixing models are highly sensitive to variation in discrimination factors. Ecological Applications. 2011;21(4):1017–23. doi: 10.1890/09-2409.1 21774408

[21] Bastos RF, Corrêa F, Winemiller KO, Garcia AM. Are you what you eat? Effects of trophic discrimination factors on estimates of food assimilation and trophic position with a new estimation method. Ecological Indicators. 2017;75:234–41.

[22] Stephens RB, Ouimette AP, Hobbie EA, Rowe RJ. Reevaluating trophic discrimination factors (Δδ13C and Δδ15N) for diet reconstruction. Ecological Monographs. 2022;92(3):e1525.

[23] Derbridge JJ, Merkle JA, Bucci ME, Callahan P, Koprowski JL, Polfus JL, et al. Experimentally derived δ13C and δ15N discrimination factors for gray wolves and the impact of prior information in Bayesian mixing models. PloS one. 2015;10(3):e0119940. 25803664 10.1371/journal.pone.0119940 PMC4372554

[24] Swan GJ, Bearhop S, Redpath SM, Silk MJ, Goodwin CE, Inger R, et al. Evaluating Bayesian stable isotope mixing models of wild animal diet and the effects of trophic discrimination factors and informative priors. Methods in Ecology and Evolution. 2020;11(1):139–49.

[25] Moore JW, Semmens BX. Incorporating uncertainty and prior information into stable isotope mixing models. Ecology letters. 2008;11(5):470–80. doi: 10.1111/j.1461-0248.2008.01163.x 18294213

[26] Hopkins JBI, Ferguson JM. Estimating the diets of animals using stable isotopes and a comprehensive Bayesian mixing model. PLoS One. 2012;7(1):e28478. doi: 10.1371/journal.pone.0028478 22235246 PMC3250396

[27] Healy K, Guillerme T, Kelly SB, Inger R, Bearhop S, Jackson AL. SIDER: An R package for predicting trophic discrimination factors of consumers based on their ecology and phylogenetic relatedness. Ecography. 2018;41(8):1393–400.

[28] Morgenthaler A, Millones A, Gandini P, Frere E. Which trophic discrimination factors fit the best? A combined dietary study of a coastal seabird. Journal of Ornithology. 2021;162:179–90.

[29] Sugden S, Murray M, Edwards MA, St. Clair CC. Inter-population differences in coyote diet and niche width along an urban–suburban–rural gradient. Journal of Urban Ecology. 2021;7(1).

[30] Fathabadi A, Jansen JD. Quantifying uncertainty of sediment fingerprinting mixing models using frequentist and Bayesian methods: A case study from the Iranian loess Plateau. Catena. 2022;217:106474.

[31] Thieltges DW, Goedknegt MA, O’Dwyer K, Senior AM, Kamiya T. Parasites and stable isotopes: a comparative analysis of isotopic discrimination in parasitic trophic interactions. Oikos. 2019;128(9):1329–39.

[32] Zuidhof M, Schneider B, Carney V, Korver D, Robinson F. Growth, efficiency, and yield of commercial broilers from 1957, 1978, and 2005. Poultry science. 2014;93(12):2970–82. doi: 10.3382/ps.2014-04291 25260522 PMC4988556

[33] Reich KJ, Bjorndal KA, Martínez del Rio C. Effects of growth and tissue type on the kinetics of 13C and 15N incorporation in a rapidly growing ectotherm. Oecologia. 2008;155(4):651–63. doi: 10.1007/s00442-007-0949-y 18188602

[34] Buchheister A, Latour RJ. Turnover and fractionation of carbon and nitrogen stable isotopes in tissues of a migratory coastal predator, summer flounder (Paralichthys dentatus). Canadian Journal of Fisheries and Aquatic Sciences. 2010;67(3):445–61.

[35] Campos D, Macari M, Fernadez-Alarcon M, Nogueira W, de Souza F, Hada F, et al. Performance and carbon turnover in fast-and slow-growing broilers submitted to cyclic heat stress and fed on high-protein diets. British poultry science. 2016;57(1):84–92. doi: 10.1080/00071668.2015.1115467 26634283

[36] Scharnweber K, Andersson ML, Chaguaceda F, Eklöv P. Intraspecific differences in metabolic rates shape carbon stable isotope trophic discrimination factors of muscle tissue in the common teleost Eurasian perch (Perca fluviatilis). Ecology and Evolution. 2021;11(14):9804–14. doi: 10.1002/ece3.7809 34306663 PMC8293782

[37] Gorokhova E. Individual growth as a non-dietary determinant of the isotopic niche metrics. Methods in Ecology and Evolution. 2018;9(2):269–77.

[38] Ogden LJE, Hobson KA, Lank DB. Blood isotopic (δ13C and δ15N) turnover and diet-tissue fractionation factors in captive dunlin (Calidris alpina pacifica). The Auk. 2004;121(1):170–7.

[39] Oortwijn T, de Monte LG, Varley DP, van der Meer MT, van Gils JA. Tissue-and diet-dependent stable carbon and nitrogen isotope discrimination: a calibration study in a captive shorebird species. Journal of Avian Biology. 2023:e03094.

[40] Stephens RB, Shipley ON, Moll RJ. Meta-analysis and critical review of trophic discrimination factors (Δ13C and Δ15N): Importance of tissue, trophic level and diet source. Functional Ecology. 2023;n/a(n/a).

[41] Aviagen. Ross 308 Nutrient Specifications 2014 [http://en.aviagen.com/assets/Tech_Center/Ross_Broiler/Ross308BroilerNutritionSpecs2014-EN.pdf.

[42] Paul D, Skrzypek G, Fórizs I. Normalization of measured stable isotopic compositions to isotope reference scales–a review. Rapid Communications in Mass Spectrometry. 2007;21(18):3006–14. doi: 10.1002/rcm.3185 17705258

[43] Bond AL, Hobson KA. Reporting stable-isotope ratios in ecology: recommended terminology, guidelines and best practices. Waterbirds. 2012;35(2):324–31.

[44] Dunn PJHC J.F. Good practice guide for isotope raito mass spectrometry. Second ed: Forensic Inotope Ratio Mass Spectrometry (FIRMS) Network; 2018 2018. 94 p.

[45] Qi H, Coplen TB, Mroczkowski SJ, Brand WA, Brandes L, Geilmann H, et al. A new organic reference material, l-glutamic acid, USGS41a, for δ13C and δ15N measurements− a replacement for USGS41. Rapid Communications in Mass Spectrometry. 2016;30(7):859–66. 26969927 10.1002/rcm.7510

[46] Song C, Kuznetsova O, editors. Fitting Gompertz Nonlinear Mixed Model to Infancy Growth Data with SAS-« version 8 Procedure NLMIXED2001 2001.

[47] Sakomura N, Longo F, Oviedo-Rondon E, Boa-Viagem C, Ferraudo A. Modeling energy utilization and growth parameter description for broiler chickens. Poultry Science. 2005;84(9):1363–9. doi: 10.1093/ps/84.9.1363 16206556

[48] Littell RC, Milliken GA, Stroup WW, Wolfinger RD, Schabenberger O. SAS system for Mixed Models, Second Edition. Second ed. Cary, NC: SAS Institute Inc.; 2006 2006.

[49] Kiernan K, Tao J, Gibbs P, editors. Tips and strategies for mixed modeling with SAS/STAT^®^ procedures. SAS Global Forum; 2012: SAS Institute Inc Cary, NC.

[50] SAS Institute Inc. SAS/STAT^®^ 9.3 User’s Guide Cary, NC: SAS Institute Inc; 2011.

[51] Burnham KR, Anderson DR. Model selection and inference: a practical information-theoretic approach. 2nd ed. New York, New York: Springer-Verlag; 2002 2002.

[52] He M, Liu F, Wang F. Determination of the stable isotope discrimination factor of wild organisms: a case study of the red swamp crayfish in integrated rice–crayfish (Procambarus clarkii) culture without artificial diets. Isotopes in Environmental and Health Studies. 2022;58(1):81–98. doi: 10.1080/10256016.2021.2008380 34890270

[53] Arnold TW. Uninformative parameters and model selection using Akaike’s information criterion. Journal of Wildlife Management. 2010;74(6):1175–8.

[54] Stock BC, Semmens BX. MixSIAR GUI user manual. Version 3.1. 2016.

[55] Spiegelhalter DJ, Best NG, Carlin BP, Linde A. The deviance information criterion: 12 years on. Journal of the Royal Statistical Society: Series B (Statistical Methodology). 2014;76(3):485–93.

[56] Hughes KL, Whiteman JP, Newsome SD. The relationship between dietary protein content, body condition, and Δ 15 N in a mammalian omnivore. Oecologia. 2018;186:357–67. 29189940 10.1007/s00442-017-4010-5

[57] Roth JD, Hobson KA. Stable carbon and nitrogen isotopic fractionation between diet and tissue of captive red fox: implications for dietary reconstruction. Canadian Journal of Zoology. 2000;78(5):848–52.

[58] Whiteman JP, Kim SL, McMahon KW, Koch PL, Newsome SD. Amino acid isotope discrimination factors for a carnivore: physiological insights from leopard sharks and their diet. Oecologia. 2018;188(4):977–89. 30349939 10.1007/s00442-018-4276-2

[59] Rosenfeld J, Van Leeuwen T, Richards J, Allen D. Relationship between growth and standard metabolic rate: measurement artefacts and implications for habitat use and life‐history adaptation in salmonids. Journal of Animal Ecology. 2015;84(1):4–20. doi: 10.1111/1365-2656.12260 24930825

[60] Hobson KA, Clark RG. Assessing avian diets using stable isotopes.2. Factors influencing diet-tissue fractionation. Condor. 1992;94(1):189–97.

[61] Coletta L, Pereira A, Coelho A, Savino VJM, Menten JFM, Correr E, et al. Barn vs. free-range chickens: Differences in their diets determined by stable isotopes. Food Chemistry. 2012;131(1):155–60.

[62] Micklem IA, Connan M, Stander N, McQuaid CD. Influence of ontogeny on stable isotope ratios and trophic discrimination factors of African penguin (Spheniscus demersus) tissues. Marine Biology. 2021;168(7):96.

[63] Cruz V, Ducatti C, Pezzato AC, Pinheiro D, Sartori JR, Goncalves J, et al. Influence of diet on assimilation and turnover of 13C in the tissues of broiler chickens. British poultry science. 2005;46(3):382–9. doi: 10.1080/0071660500126847 16050194

[64] Pelícia V, Araujo P, Luiggi F, Stradiotti A, Denadai J, Sartori J, et al. Estimation of the metabolic rate by assessing carbon-13 turnover in broiler tissues using the stable isotope technique. Livestock Science. 2018;210:8–14.

[65] Wolf N, Newsome SD, Peters J, Fogel ML. Variability in the routing of dietary proteins and lipids to consumer tissues influences tissue-specific isotopic discrimination. Rapid Communications in Mass Spectrometry. 2015;29(15):1448–56. doi: 10.1002/rcm.7239 26147485

[66] McMahon KW, Polito MJ, Abel S, McCarthy MD, Thorrold SR. Carbon and nitrogen isotope fractionation of amino acids in an avian marine predator, the gentoo penguin (Pygoscelis papua). Ecology and Evolution. 2015;5(6):1278–90. doi: 10.1002/ece3.1437 25859333 PMC4377271

[67] Whiteman JP, Rodriguez Curras M, Feeser KL, Newsome SD. Dietary protein content and digestibility influences discrimination of amino acid nitrogen isotope values in a terrestrial omnivorous mammal. Rapid Communications in Mass Spectrometry. 2021;35(11):e9073. doi: 10.1002/rcm.9073 33634533

[68] Kurle CM, Koch PL, Tershy BR, Croll DA. The effects of sex, tissue type, and dietary components on stable isotope discrimination factors (Δ13C and Δ15N) in mammalian omnivores. Isotopes in Environmental and Health Studies. 2014;50(3):307–21. 24787278 10.1080/10256016.2014.908872

[69] Lecomte N, Ahlstrom O, Ehrich D, Fuglei E, Ims RA, Yoccoz NG. Intrapopulation variability shaping isotope discrimination and turnover: experimental evidence in Arctic foxes. PLoS One. 2011;6(6). doi: 10.1371/journal.pone.0021357 21731715 PMC3121787

[70] Johnston TA, Casselman JM, Leggett WC. Sex-based divergence in isotopic compositions of north temperate freshwater fishes. Hydrobiologia. 2021;848(4):873–84.

[71] Caut S, Angulo E, Courchamp F, Figuerola J. Trophic experiments to estimate isotope discrimination factors. Journal of Applied Ecology. 2010;47(4):948–54.

[72] McMahon KW, Fogel ML, Elsdon TS, Thorrold SR. Carbon isotope fractionation of amino acids in fish muscle reflects biosynthesis and isotopic routing from dietary protein. Journal of Animal Ecology. 2010;79(5):1132–41. doi: 10.1111/j.1365-2656.2010.01722.x 20629794

[73] Ishikawa NF. Use of compound-specific nitrogen isotope analysis of amino acids in trophic ecology: assumptions, applications, and implications. Ecological Research. 2018;33(5):825–37.

[74] Petta JC, Shipley ON, Wintner SP, Cliff G, Dicken ML, Hussey NE. Are you really what you eat? Stomach content analysis and stable isotope ratios do not uniformly estimate dietary niche characteristics in three marine predators. Oecologia. 2020;192(4):1111–26. doi: 10.1007/s00442-020-04628-6 32179976

[75] Davis P, Syme J, Heikoop J, Fessenden-Rahn J, Perkins G, Newman B, et al. Quantifying uncertainty in stable isotope mixing models. Journal of Geophysical Research: Biogeosciences. 2015;120(5):903–23.

[76] Tarroux A, Ehrich D, Lecomte N, Jardine TD, Bêty J, Berteaux D. Sensitivity of stable isotope mixing models to variation in isotopic ratios: evaluating consequences of lipid extraction. Methods in Ecology and Evolution. 2010;1(3):231–41.

[77] Cloyed CS, DaCosta KP, Hodanbosi MR, Carmichael RH. The effects of lipid extraction on δ13C and δ15N values and use of lipid-correction models across tissues, taxa and trophic groups. Methods in Ecology and Evolution. 2020;11(6):751–62.

[78] Pagani-Núñez E, Barnett CA, Gu H, Goodale E. The need for new categorizations of dietary specialism incorporating spatio-temporal variability of individual diet specialization. Journal of Zoology. 2016;300(1):1–7.

[79] Maldonado K, Bozinovic F, Newsome SD, Sabat P. Testing the niche variation hypothesis in a community of passerine birds. Ecology. 2017;98(4):903–8. doi: 10.1002/ecy.1769 28170088

[80] Schulting RJ, MacDonald R, Richards MP. FRUITS of the sea? A cautionary tale regarding Bayesian modelling of palaeodiets using stable isotope data. Quaternary International. 2023;650:52–61.

[81] Litmanen JJ, Perälä TA, Taipale SJ. Comparison of Bayesian and numerical optimization-based diet estimation on herbivorous zooplankton. Philosophical Transactions of the Royal Society B: Biological Sciences. 2020;375(1804):20190651. doi: 10.1098/rstb.2019.0651 32536310 PMC7333965

