# Supplementary material for: Testing for effects of growth rate on isotope trophic discrimination factors and evaluating the performance of Bayesian stable isotope mixing models experimentally: A moment of truth?
Source: PLoS One. 2024 Jun 14;19(6):e0304495. doi: 10.1371/journal.pone.0304495 (PMC11178173; doi:10.1371/journal.pone.0304495)
Supplement: S1 Table — Percentages of each constituent are shown. (PDF) [file pone.0304495.s001.pdf]

S1 Table. Commercially available feed ingredients (by percentage) used to formulate isotopically distinct, nutritionally-balanced diets for as-hatched Ross 308 broiler chicks.

Ingredient	Diet 1	Diet 2	Diet 3	Diet 4
Corn	59.86	0.00	40.10	19.75
Wheat	0.00	58.85	19.42	39.43
Soybean meal	16.82	31.49	21.66	26.65
Corn gluten meal	9.00	0.01	6.03	2.97
Fish meal	10.00	0.00	6.70	3.30
Canola oil	1.00	4.84	2.27	3.57
Limestone	1.09	1.50	1.23	1.36
Mono-dicalcium phosphate	0.21	1.20	0.54	0.87
Sodium chloride	0.02	0.38	0.14	0.26
L-arginine	0.44	0.00	0.29	0.15
DL-methionine	0.16	0.31	0.21	0.26
L-lysine - HCL	0.23	0.16	0.21	0.18
L-threonine	0.06	0.16	0.09	0.13
Ameri-Bond 2x[a]	0.50	0.50	0.50	0.50
Econase[b]	0.01	0.01	0.01	0.01
Broiler vitamin / mineral premix[c]	0.50	0.50	0.50	0.50
Choline chloride	0.10	0.10	0.10	0.10

[a] (Pellet binder; LignoTech, Wisconsin, USA)

[b] (Econase XT; ABVista, Wiltshire, UK, β 1-4 endo-xylanase enzyme, xylanase activity; 160,000 BXU g^{-1})

[c] (Supplied per kilogram of diet: vitamin A (retinyl acetate + retinyl palmitate), 11000 IU; vitamin d3, 2200 IU; vitamin E (dl-α-tocopheryl acetate), 300 IU; menadione, 2.0 mg; thiamine, 1.5 mg; riboflavin, 6.0 mg; niacin, 60 mg; pyridoxine, 4 mg; vitamin B12,
