# Supplementary material for: Testing for effects of growth rate on isotope trophic discrimination factors and evaluating the performance of Bayesian stable isotope mixing models experimentally: A moment of truth?
Source: PLoS One. 2024 Jun 14;19(6):e0304495. doi: 10.1371/journal.pone.0304495 (PMC11178173; doi:10.1371/journal.pone.0304495)
Supplement: Attachment — Submitted filename: Response to Reviewers.docx [file pone.0304495.s002.docx]

Dr. Lee W. Cooper

Section Editor, PLOS ONE

University of Maryland

Solomons, Maryland

May 7, 2024

Dear Dr. Cooper;

We are grateful for an opportunity to submit a revised version of our manuscript, “Testing for effects of growth rate on isotope trophic discrimination factors and evaluating the performance of Bayesian stable isotope mixing models experimentally: a moment of truth?” (PONE-D-24-03114), by K. Gurney et al. The review comments have helped us to improve the clarity and rigour of the manuscript. Based on your most recent correspondence (01 April 2024), we understand that main concerns relate to:

(1) compliance with journal requirements (as below), and
(2) statistical comparisons of mixing model results.

We discuss each of these issues in detail when responding to each individual comment (in italics, below), but highlights of our responses are also provided here, for summary purposes:

(1) To ensure that we are in strict compliance with journal formatting and data sharing requirements, we have revised text throughout the document, as requested. We have also uploaded our data to Zenodo and published it, so that it is now publicly available.
(2) We have revised the statistical approach as suggested by Reviewer 2 and have made modifications to the Tables (and captions) that we hope will make them more reader-friendly.

**Journal Requirements:**

When submitting your revision, we need you to address these additional requirements.

1. Please ensure that your manuscript meets PLOS ONE's style requirements, including those for file naming.

- *Response: We have carefully reviewed the style templates and made every effort to ensure that the revision is formatted in line with PLOS ONE’s requirements. If further formatting changes are required, we can make those changes quickly.*

2. Thank you for stating the following financial disclosure:

Environment and Climate Change Canada

Please state what role the funders took in the study. If the funders had no role, please state: "The funders had no role in study design, data collection and analysis, decision to publish, or preparation of the manuscript."

If this statement is not correct you must amend it as needed. Please include this amended Role of Funder statement in your cover letter; we will change the online submission form on your behalf.

- *Response: Funding was provided by Environment and Climate Change Canada. The funders had no role in study design, data collection and analysis, decision to publish, or preparation of the manuscript.*

3. Thank you for uploading your study's underlying data set. Unfortunately, the repository you have noted in your Data Availability statement does not qualify as an acceptable data repository according to PLOS's standards.

At this time, please upload the minimal data set necessary to replicate your study's findings to a stable, public repository (such as figshare or Dryad) and provide us with the relevant URLs, DOIs, or accession numbers that may be used to access these data. For a list of recommended repositories and additional information on PLOS standards for data deposition, please see <https://journals.plos.org/plosone/s/recommended-repositories>.

- *Response: Thank you for sharing the link to the recommended repositories. We have uploaded our complete data sets to the Zenodo general-purpose open repository. The DOI that can be used to access these data is* [*https://doi.org/10.5281/zenodo.10927544*](https://doi.org/10.5281/zenodo.10927544)

4. When completing the data availability statement of the submission form, you indicated that you will make your data available on acceptance. We strongly recommend all authors decide on a data sharing plan before acceptance, as the process can be lengthy and hold up publication timelines. Please note that, though access restrictions are acceptable now, your entire data will need to be made freely accessible if your manuscript is accepted for publication. This policy applies to all data except where public deposition would breach compliance with the protocol approved by your research ethics board. If you are unable to adhere to our open data policy, please kindly revise your statement to explain your reasoning and we will seek the editor's input on an exemption. Please be assured that, once you have provided your new statement, the assessment of your exemption will not hold up the peer review process.

- *Response: As requested, our entire data set is now freely accessible on the Zenodo general-purpose open repository (*[*https://doi.org/10.5281/zenodo.10927544*](https://doi.org/10.5281/zenodo.10927544)*) .*

5. Please include your full ethics statement in the ‘Methods’ section of your manuscript file. In your statement, please include the full name of the IRB or ethics committee who approved or waived your study, as well as whether or not you obtained informed written or verbal consent. If consent was waived for your study, please include this information in your statement as well.

- *Response: The full ethics statement is included in the ‘Methods’ section of the revised manuscript (lines 149–151) and has been modified slightly to include the names of both the Institutional Research Board (i.e., Animal Research Ethics) and the Committee (i.e., Animal Care Committee), which provided written approval of the study.*

6. We note that you have referenced Campos D, Macari M, Fernadez-Alarcon M, Nogueira W, de Souza F, Hada F, et al. which has currently not yet been accepted for publication. Please remove this from your References and amend this to state in the body of your manuscript: (Campos D, Macari M, Fernadez-Alarcon M, Nogueira W, de Souza F, Hada F, et al. [Submitted]) as detailed online in our guide for authors

- *Response: With apologies for my confusion, I am uncertain as to why this article is considered as not yet accepted for publication? I find that it was published in the British Poultry Science journal (January 2016) and that the open access article (DOI: 10.1080/00071668.2015.1115467; ISSN 00071668) is listed both in Web of Science and Scopus.*
- *If there is further concern with this reference, please advise.*

7. Please review your reference list to ensure that it is complete and correct. If you have cited papers that have been retracted, please include the rationale for doing so in the manuscript text, or remove these references and replace them with relevant current references. Any changes to the reference list should be mentioned in the rebuttal letter that accompanies your revised manuscript. If you need to cite a retracted article, indicate the article’s retracted status in the References list and also include a citation and full reference for the retraction notice.

- *Response: The reference list has been carefully reviewed and is complete and correct.*

**Reviewers' comments to the Author**

Lines 55-56: I don’t think inert tissues are necessarily sampled non-invasively. For example, plucking a feather or clipping a nail is considered invasive sampling.

- *Response: We have modified this sentence to state that the sampling of inert tissues is non-lethal and less invasive than sampling of metabolically active tissues (lines 55–57).*

Lines 184-185: It doesn’t appear that the many of the measured d15N values ended up falling within the range of the values of the standards. What influence might this have on the data?

- *Response: We agree that it is ideal to have the secondary reference materials bracket sample isotopic compositions, but this can be challenging to achieve in all cases. Studies that have reviewed this issue (listed below) indicate that secondary reference materials that do not bracket the experimental values do not have a statistically meaningful influence on the data, as calibration – especially for carbon and nitrogen – most commonly remains linear beyond the range of the used reference materials.*
  - *Bond, Alexander L., and Keith A. Hobson. "Reporting stable-isotope ratios in ecology: recommended terminology, guidelines and best practices." Waterbirds 35.2 (2012): 324-331.*
  - *Carter, J. F., and Brian Fry. "Ensuring the reliability of stable isotope ratio data—beyond the principle of identical treatment." Analytical and Bioanalytical Chemistry 405 (2013): 2799-2814.*

Lines 170-193: I was expecting this section to also detail how the isotope data were normalized to the international scale, but that was missing. Please add those details.

- *Response: Thank you for the suggestion. We have added text (lines 185–186 and lines 189–192), further describing the normalization and calibration methods used by the analytical lab.*

Lines 378-379: Although there is a statistical effect of sex on TDF for d13C, the size of this effect (which appears to be <0.3 per mil) is quite small, which seems worth saying somewhere in this paragraph.

- *Response: We note that the effect of sex on TDF was observed for d15N, rather than d13C, but have modified the paragraph to highlight the small effect size for sex (line 399).*

Discussion: I didn’t feel that the Discussion completely addressed this accuracy-related question that was stated on lines 107-108. Please consider revising the Discussion to do so.

- *Response: Thank you. Based on feedback from Reviewer 2, we have revised the manuscript content to include more information on assessing and describing accuracy (see lines 236–240 and 327–332; Tables 4 and 5).*
- *We also refer back to these descriptions when discussing model performance, especially lines 435–445, where we note that relative dietary contributions were similar to known proportions. We hope that these adjustments address the Reviewer’s concern.*

**Reviewer #2:** This study is original and well designed; and the manuscript is well written. I should be acceptable for publication following minor revisions. Specifically, while most of the statistical analyses used in the manuscript is appropriate, I suggest that authors revise their reporting and comparisons of mixing model results across feeding groups. The current approach uses qualitative comparisons and the mean and SD reporting of model results in Tables 4 and 5 makes it challenging for the reader to understand what is being reported in these tables. Specific recommendations are outlined below:

Line 228-229: This section described how models with different terms/structures were ranked using DIC but does not include text on how the results of these model quantitatively were compared across diet groups. Based on Figure 5, it appears that this was done in qualitative manner by plotting the actual diet proportions for each group on mixing model posterior distribution plots. I recommend that the authors formalize and describe their approach to comparing model results here in the methods.

Specifically, I would recommend that qualitatively compare the actual diet proportions for each group to the 95% CI resulting from each mixing model. If the actual falls within the 95%CI then it is “similar” (i.e., mixing model is accurate) but if it does not than they are different (i.e., mixing model is not accurate).

- *Response: We are grateful for this reviewer’s suggestions for improving interpretation of model outputs. We now provide 95% credible intervals (and medians) for all model outputs (Tables 4 and 5). We also highlight in these tables where the credible interval includes the known proportions – for the best approximating model (both tissues).*
- *To describe these changes, we have added text to the methods (lines 236–239) and have also added more details to highlight our findings in the results (lines 327–332). We also make reference to the revised tables in the Discussion when mentioning that accuracy of the model outputs (rather than inference) is affected more by model specifications (line 435–437).*

Table 4 & 5: I find these tables very hard to interpret even with the sub-caption. Also it is uncommon to report the results of Bayesian mixing models as Mean±SD. Reporting mixing model results as means ± SDs can lead to biased interpretations and fundamentally does not align with the probabilistic concepts underlying the Bayesian statistics used in these models. While others have reported Bayesian mixing model results in this manner it does not mean that doing so is always appropriate. This is because MixSIAR estimates proportions (that must sum to 1) they are almost always skewed (not a normal distribution). In addition, they can also be multimodal (though that does not appear to be the case in this dataset).

That said the posterior distribution plots provided in Figure 5 indicate some long tails that are better accounted for with median and 95%CI intervals provided by MixSIAR. Therefore, please report mixing model results as median values with upper and lower 95% credibility intervals in Table 4 and 5. This will also facilitate the more quantitative comparison between diet groups recommended above.

- *Response: Again, we thank the reviewer for this feedback and have made efforts to clarify the tables through improved captions. Also, as indicated previously, both tables now include median and 95% credible intervals for all model outputs. Please see lines 327–332 for the more quantitative comparison between diet groups.*

**Other comments:**

- Avoid “isotopic signatures” and use “isotopic values” instead.

- *Response: Changes (x4) as suggested. Please see lines 73, 102, 114, and 368.*

- Line 173: lipid extraction can bias d15N values in tissues, which can in turn bias mixing model results.

(Tarroux, A., Ehrich, D., Lecomte, N., Jardine, T. D., Bêty, J., & Berteaux, D. (2010). Sensitivity of stable isotope mixing models to variation in isotopic ratios: evaluating consequences of lipid extraction. Methods in Ecology and Evolution, 1(3), 231-241.).

Please clarify how this might have affected your results and address this in the discussion.

- *Response: Although the effects of lipid extraction on estimated dietary proportions by BSIMMs in this study are not known, we note that (i) all samples were treated (i.e. extracted) similarly using established methods, and (ii) all diets had similar C:N ratios, such that effect of lipid extraction on δ^13^C of food samples should have been similar across groups, with minimal expected effect on δ^15^N for most tissues (egg being an exception, which we did not use), as described by Tarroux et al. (see pages 232 and 237).*
- *Tarroux et al. also find (see page 237) that when lipid extraction of a consumer tissue strongly effects its isotopic values (i.e., higher lipid content in the bulk tissue), not lipid extracting that tissue can lead to incorrect conclusions about diet. For this reason, we consider that extraction of lipids from liver (a relatively lipid-rich tissue) would have been unlikely to invalidate our estimates of diet composition. Feather tissues, conversely, do not contain lipids, so the potential for an effect of using lipid-extracted isotope values for food items on BSIMMs outputs would be higher; however, estimates of accuracy for both liver and feather tissues were similar, suggesting that this was not the case.*

- Line 214: Why 85% and not 95%?

- *Response: As noted in the cited reference (Arnold 2010, full citation shown below), the use of 85% confidence intervals is more compatible with model selection using information theory, resulting in reduced ambivalence with respect to selecting informative parameters. This rationale is highlighted at lines 221–224.*
  - *Arnold TW. Uninformative parameters and model selection using Akaike's information criterion. Journal of Wildlife Management. 2010;74(6):1175-8.*
